# Retraction Note: CT, MRI, and 18F-FDG PET/CT imaging features of seven cases of adult pancreatoblastoma

**DOI:** 10.1186/s12880-024-01298-1

**Published:** 2024-05-10

**Authors:** Mengnan Wu, Jiongbin Lin, Zhuangsheng Liu, Zhiming Huang, Ruoning Wang

**Affiliations:** 1 Department of Radiology, Heyuan People’s Hospital, General Hospital Guangdong, Heyuan, 517000 China; 2 Department of Radiology, Nanfang Hospital, Southern Medical University, Guangzhou, 510515 China; 3 Department of Radiology, The Sixth Affiliated Hospital, South China University of Technology, Nanhai People’s Hospital, Foshan, 528200 Guangdong China; 4 Minimally Invasive Centre, Tumour Hospital, Sun Yat-Sen University, Guangzhou, 510060 China

**Retraction Note: BMC Medical Imaging 22, 228 (2022)**

**https://doi.org/10.1186/s12880-022-00958-4**

The Editor has retracted this article. After publication, concerns were raised about the provenance of some of the data, which the authors have not been able to satisfactorily address. The Editor therefore no longer has confidence in the results and conclusions presented.

Mengnan Wu does not agree with this retraction. Jiongbin Lin, Zhuangsheng Liu, Zhiming Huang & Ruoning Wang have not responded to correspondence from the editor about this retraction.

